# Mechanisms and management of pegylated interferon-α toxicity in chronic hepatitis B

**DOI:** 10.3389/fimmu.2026.1801043

**Published:** 2026-04-10

**Authors:** Liya Zhu, Fei Peng, Dingfang Pi, Jinzhi Lu

**Affiliations:** 1 Department of Infectious Diseases, The First Affiliated Hospital of Yangtze University, Jingzhou, Hubei, China; 2 Department of Laboratory Medicine, The First Affiliated Hospital of Yangtze University, Jingzhou, Hubei, China

**Keywords:** adverse drug reactions, chronic hepatitis B, functional cure, immune dysregulation, JAK-STAT pathway, pegylated interferon-α, systemic immune activation, treatment management

## Abstract

Pegylated interferon-α (PegIFN-α) is a cornerstone immunomodulatory therapy with the potential to achieve a functional cure for chronic hepatitis B (CHB). However, its clinical application is constrained by a high frequency of multisystem adverse reactions, which often lead to dose modifications or treatment discontinuation. These toxicities are not ancillary effects but rather direct consequences of the drug’s therapeutic mechanism, arising from a broad and often dysregulated systemic immune activation. This review systematically delineates the clinical spectrum of these reactions and provides a unified framework for their underlying host immunopathological mechanisms. We dissect the interconnected network of host factors including cytokine-driven inflammation, hematopoietic suppression, loss of immune tolerance, and neuro-immune axis disruption that mediate common toxicities such as flu-like syndrome, cytopenias, autoimmunity, and neuropsychiatric symptoms. A focused analysis of ocular vascular injury is included. By synthesizing recent evidence, we discuss predictive biomarkers and contemporary management strategies designed to navigate this therapeutic dichotomy. Finally, we outline future directions for developing safer, mechanism-informed, and host-directed personalized therapies for CHB.

## 1 Introduction

Chronic hepatitis B virus (HBV) infection remains a formidable global health challenge, contributing substantially to the burden of liver cirrhosis and hepatocellular carcinoma (1). Within the current therapeutic landscape, Nucleos(t)ide analogues (NAs) effectively suppress viral replication but achieve functional cure (sustained HBsAg loss) in only a small fraction of patients, such as 1–3% after one year, with only a slight increase after prolonged therapy (2, 3), often necessitating indefinite treatment. In contrast, pegylated interferon-α (PegIFN-α) offers a finite treatment course (48 weeks) and achieves functional cure in 3–10% of HBeAg-positive and up to 5% of HBeAg-negative patients, reaching 16% in those with favorable baseline predictors (4–6). This finite-course strategy hinges on the potent, broad activation of the host immune system to reverse the state of immune dysfunction and T-cell exhaustion that characterizes chronic HBV infection (7–9).

However, this specific property of systemic immunostimulation embodies a profound immunological paradox. The exact mechanisms that drive viral clearance are inseparable from those that cause multisystem toxicity (7, 8). PegIFN-α therapy triggers immunomodulatory events that can result in divergent clinical outcomes, ranging from clearance of infected hepatocytes to collateral tissue damage (7, 8). These opposing outcomes represent different manifestations of the same activated immune state, a precarious balance that stems from the fundamental challenge of discriminating between viral and self-antigens within a systemically activated and often dysregulated immune landscape (9). Critically, the immunostimulatory program driven by PegIFN-α is not unidirectional; it can simultaneously engage counter-regulatory circuits that actively suppress antiviral immunity (10–13). This dual capacity is rooted in the fundamental role of type I interferon in immune homeostasis and autoinflammation (10). It is a recognized phenomenon in chronic infection models (11) and has been directly demonstrated in patients, where therapy induces specific immunosuppressive cell populations that curtail treatment efficacy (12). This activated state not only risks collateral tissue damage but can also paradoxically deplete the very antiviral effectors it seeks to mobilize, such as CD8^+^ T cells, resulting in immune activation paradoxically coupled with lymphocyte consumption (13). Consequently, the JAK-STAT pathway, central to PegIFN-α signaling, mediates both the desired antiviral response and a frequent array of adverse reactions, reflecting the divergent outcomes of systemic immune activation in different cellular contexts (6–8).

This review therefore aims to synthesize current knowledge by proposing and elaborating a unified framework of systemic immune activation and dysregulation that explains PegIFN-α-induced toxicities. We provide a structured analysis spanning clinical presentation to molecular pathogenesis and outline evolving strategies for prediction and personalized management, including monitoring for specific end-organ damage. This framework directly addresses the core efficacy-toxicity paradox of interferon therapy by elucidating the pathways through which a single immunostimulatory trigger leads to divergent clinical outcomes.

## 2 Clinical manifestations of adverse reactions

The adverse effect profile of PegIFN-α is extensive and well-characterized, a direct reflection of its systemic immunomodulatory action. Clinically, these manifestations represent the spectrum of immune hyperactivation and loss of specificity.

The most common complaints are an initial, self-limiting flu-like syndrome (fever, chills, myalgia, headache) and a more persistent, debilitating fatigue, which affect the majority of patients and frequently necessitate symptomatic management (e.g., with analgesics) or dose modification (14–17). Hematological toxicity, primarily neutropenia and thrombocytopenia, constitutes a major dose-limiting concern, with an incidence ranging broadly from 20% to 80%. While severe neutropenia (absolute neutrophil count [ANC] <0.75 × 10^9^/L) is less frequent, it represents a leading cause for dose modification or interruption (7, 14). The pathogenesis is multifactorial (18), and these cytopenias significantly increase the risk of infection and bleeding (14).

Neuropsychiatric effects, such as depression, irritability, and insomnia, are particularly debilitating. They affect an estimated 15–30% of patients, with major depression occurring in approximately 5% or less, and require vigilant monitoring due to their severe impact on treatment adherence and patient well-being (19). Autoimmune thyroid dysfunction is the most prevalent immune-mediated adverse reaction during PegIFN-α therapy. Recent longitudinal studies report an incidence of 8.8–15.8%, with a characteristic biphasic pattern in which an initial destructive thyrotoxicosis (transient, often subclinical hyperthyroidism) occurring in the first 3–6 months of therapy is followed by progression to permanent hypothyroidism in a subset of patients (20, 21). This evolution reflects the underlying immunopathogenesis, in which PegIFN-α triggers a type I interferon-driven break in immune tolerance, leading to lymphocytic infiltration of the thyroid, cytokine-mediated thyrocyte injury, and subsequent anti-thyroid peroxidase (TPO) and anti-thyroglobulin (Tg) autoantibody production (22–24). Clinically, most cases are subclinical (normal free T4 with suppressed or elevated TSH), but 2–5% of patients develop overt thyroid dysfunction requiring treatment (21).

Other notable and frequently reported reactions include gastrointestinal disturbances (e.g., nausea, anorexia) and dermatological effects (e.g., alopecia, injection-site reactions), which reflect the broad systemic impact of this immune dysregulation (25). Special populations demand specific attention; for example, reversible growth retardation observed in pediatric cohorts underscores the need for careful monitoring in this group (26).

Ocular vascular toxicity presents a unique clinical picture. Prospective screening studies in patients receiving interferon therapy (for conditions ranging from chronic hepatitis to melanoma) consistently report a high incidence of retinal changes (e.g., cotton-wool spots), with the vast majority of these cases being asymptomatic (27–29). In contrast, symptomatic, sight-threatening complications (e.g., retinopathy, hemorrhage) are rare in routine practice (<1–2% of cases) (30). This marked disparity between prevalent subclinical retinal findings (e.g., cotton-wool spots in >75% of screened patients) and infrequent visual symptoms (<2%) underscores the rationale for risk-based ophthalmologic monitoring. Such an approach reserves routine fundoscopic screening for patients with vascular risk factors (hypertension, diabetes) or those reporting visual disturbances, rather than applying universal screening to all recipients of PegIFN-α therapy (29–31).

The diverse clinical manifestations, along with their estimated incidence, and key references, are comprehensively summarized in **Table 1**. This broad spectrum of toxicities raises a pivotal question of what common immunological impetus can simultaneously drive such disparate forms of tissue injury. The answer lies in a fundamental paradox at the heart of PegIFN-α therapy.

**Table 1:** Spectrum and incidence of PegIFN-α–associated adverse reactions in chronic hepatitis B.

Adverse reaction category	Specific manifestations	Estimated incidence	Subtype considerations (PegIFN-α-2a vs. -2b)	Key supporting references
Flu-like Syndrome	Fever, chills, myalgia, headache, profound fatigue	60–80%	No major differences reported; both subtypes exhibit comparable incidence and severity as a class effect (32–35).	(15–17, 36–38)
Hematological Toxicity	Neutropenia, thrombocytopenia, anemia	20–80% (Dose-dependent) [Severe neutropenia (ANC <0.75×10^9^/L) is a key reason for dose reduction]	Generally comparable between subtypes. Some evidence from HCV studies suggests a slightly higher incidence of grade 3/4 neutropenia with PegIFN-α-2b, potentially due to its wider volume of distribution and higher tissue exposure (34).	(14, 15, 18, 39–44)
Paradoxical Lymphocyte Consumption	Decreased absolute counts of circulating CD8^+^ T cells (total & virus-specific).	Commonly observed.	Insufficient comparative data; the phenomenon has been documented primarily with PegIFN-α-2a (13), but is presumed to be a class effect.	(13)
Neuropsychiatric Effects	Depression, irritability, insomnia, cognitive impairment, fatigue	15–30% (Major depression: ~≤5%)	Comparable incidence between subtypes, as demonstrated in large HCV cohort studies (33, 34).	(15, 16, 19, 45–49)
Autoimmune Phenomena	Thyroid dysfunction (hypo-/hyperthyroidism), autoantibody production (e.g., anti-TPO).	~8.8–15.8% during therapy.	Limited comparative data; both subtypes can induce thyroid dysfunction, and the incidence appears similar (32, 35).	(20–24, 50–53)
Gastrointestinal & Dermatological	GI: Nausea, diarrhea, anorexia. Derm: Alopecia, injection-site reactions, psoriasiform eruptions.	GI: 15–40%. Derm: Highly variable; alopecia is frequent (~48%).	Generally comparable. Injection-site reactions may be more frequent with PegIFN-α-2b in some reports, but data are inconsistent (35).	(15, 16, 25, 54–60)
Ocular Toxicity (Vascular)	Retinopathy (cotton-wool spots), retinal hemorrhage.	Symptomatic: Rare (<1–2%). Subclinical (on screening): 18% to >75%.	No comparative studies available. Both subtypes are associated with retinopathy, as documented in case series (29).	(27–31, 61, 62)
Hepatotoxicity (Immune-Mediated Flare)	ALT/AST elevation.	10–30%	No evidence of differential risk; flares are considered a class effect reflecting therapeutic immune activation (63, 64).	(14, 16, 17, 55, 63, 65–67)
Pulmonary Toxicity	Dyspnea, cough, interstitial pneumonitis, pulmonary arterial hypertension (PAH).	Uncommon but serious. PAH incidence low (<1–2%).	Individual case reports exist for both subtypes; no comparative data available.	(68–73)
Cardiovascular Toxicity	Arrhythmias, myopericarditis, reversible cardiomyopathy, endothelial dysfunction.	Rare *de novo* in patients with normal baseline function.	Insufficient data to compare subtypes; both are associated with rare cardiovascular events (74).	(74–82)
Renal Toxicity	Proteinuria, hematuria, acute kidney injury. Includes collapsing FSGS and thrombotic microangiopathy (TMA).	Uncommon.	Both subtypes have been implicated in case reports of glomerulopathies and TMA (83, 84).	(83–91)
Musculoskeletal & Other Systemic	Myalgia, arthralgia, exacerbation or *de novo* autoimmune rheumatic disease.	Myalgia/arthralgia: Very common. Autoimmune: Rare.	No subtype-specific differences reported.	(22, 37, 92)
Effects in Special Populations	Reversible growth retardation (in pediatric patients).	Variable (population-dependent).	Most pediatric studies have utilized PegIFN-α-2b; data for PegIFN-α-2a in children are more limited (26, 93).	(26)
Baseline Immune Dysfunction (Therapeutic Target, Not an Adverse Reaction)	T-cell exhaustion: Persistence of virologic non-response.	Variable (in non-responders).	This is a pre-existing host condition, not a drug-specific effect; the goal of reversing exhaustion applies equally to both subtypes (7, 12, 94).	(7, 12, 94)

The incidence of hematological toxicity is highly variable. Severe neutropenia (ANC <0.75 × 10^9^/L) is a primary indication for dose reduction. It is crucial to distinguish between PegIFN-α-induced toxicities (e.g., cytopenias) which are adverse consequences of systemic immune activation, and the pre-existing state of T-cell exhaustion, which is the dysfunctional immune condition characteristic of chronic HBV infection that therapy aims to reverse. The “Lymphocyte Consumption” entry describes a therapy-induced depletion, while the “Baseline Immune Dysfunction” entry describes the pre-therapy state that may persist due to inadequate reversal.

ALT, alanine aminotransferase; ANC, absolute neutrophil count; anti-TPO, anti-thyroid peroxidase antibody; AST, aspartate aminotransferase; CD8, cluster of differentiation 8; FSGS, focal segmental glomerulosclerosis; HBV, hepatitis B virus; PAH, pulmonary arterial hypertension; PegIFN-α, pegylated interferon-alpha; TMA, thrombotic microangiopathy.

## 3 Mechanisms of PegIFN-α-induced toxicity: a host immune perspective

**Figure 1:**
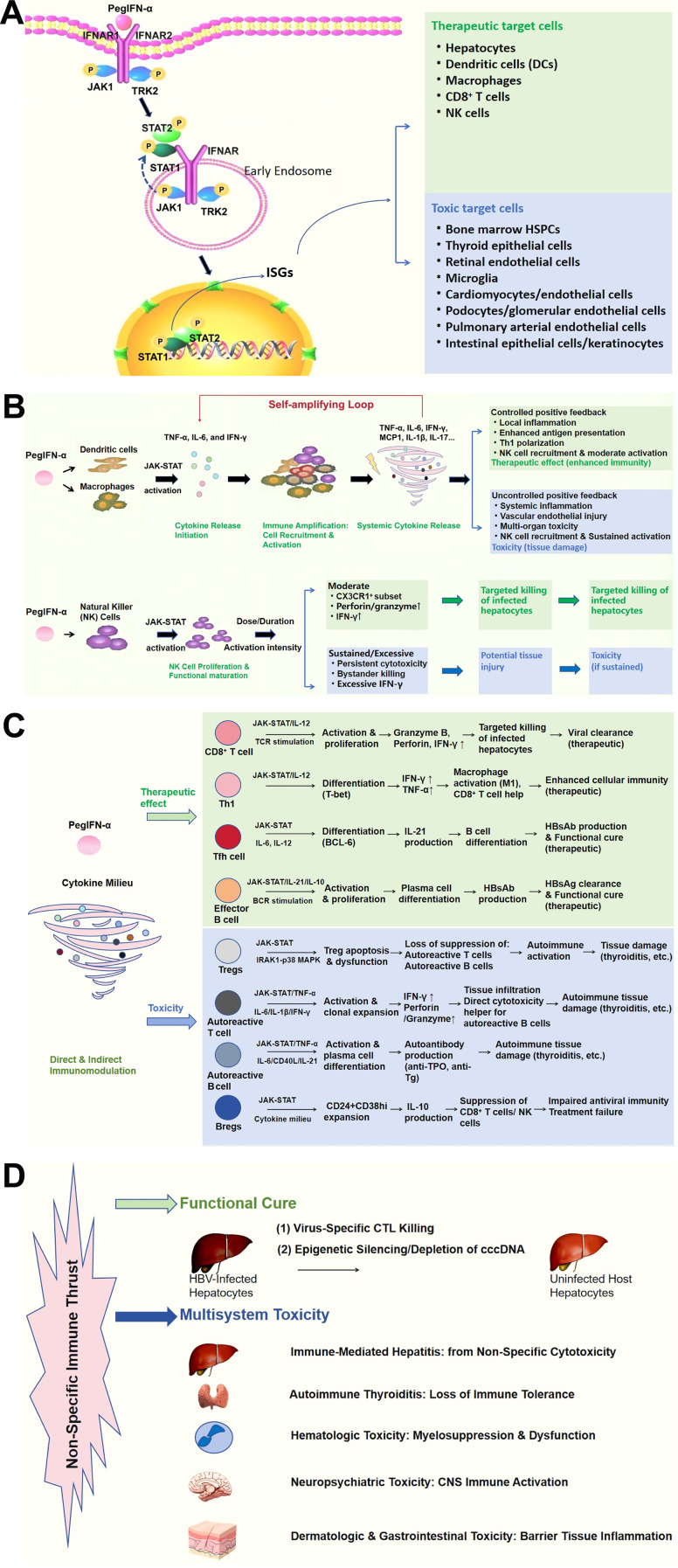
PegIFN-α-induced systemic immune activation: a unified immunological cascade driving antiviral efficacy and multisystem toxicity in chronic hepatitis B . **(A)** Molecular initiation and signaling bifurcation. PegIFN-α binds to IFNAR and activates JAK-STAT signaling, inducing ISG transcription. This signaling cascade bifurcates by cell type. Therapeutic targets include hepatocytes, dendritic cells, macrophages, HBV-specific CD8+ T cells, and NK cells. Toxic targets include bone marrow HSPCs, thyroid epithelium, retinal endothelium, microglia, cardiomyocytes and cardiac endothelium, podocytes and glomerular endothelium, pulmonary arterial endothelium, and intestinal epithelium and keratinocytes. **(B)** Innate immune amplification and divergent outcomes. In macrophages and dendritic cells, JAK-STAT activation induces cytokine release, including TNF-α, IL-6, and IFN-γ. Under controlled conditions, these local cytokines enhance antigen presentation, promote Th1 polarization, and recruit NK cells, contributing to therapeutic effects. When uncontrolled, systemic amplification of these cytokines causes flu-like syndrome, vascular injury, and multi-organ toxicity. NK cells are activated by PegIFN-α, IL-12, and IL-15. Moderate activation promotes targeted cytotoxicity and IFN-γ production, supporting antiviral immunity. Sustained activation leads to bystander killing and excessive IFN-γ production, exacerbating tissue injury. **(C)** Adaptive immunity remodeling and divergent pathways. Driven by PegIFN-α and cytokines such as IL-12, IL-6, TNF-α, and IFN-γ, adaptive immune cells diverge into therapeutic and toxic pathways. On the therapeutic side, HBV-specific CD8+ T cells eliminate infected hepatocytes through granzyme B, perforin, and IFN-γ. Tfh cells promote HBsAb production via IL-21. Th1 cells enhance antiviral immunity. Effector B cells produce HBsAb. On the toxic side, Treg apoptosis disrupts immune tolerance. Autoreactive T and B cells cause tissue damage through anti-TPO and anti-Tg antibodies. Regulatory B cells suppress CD8+ T and NK cell function via IL-10, impairing antiviral immunity. **(D)** Divergent clinical outcomes. The lack of antigenic specificity leads to two distinct clinical outcomes. When immune responses are focused on infected hepatocytes, functional cure can be achieved, characterized by HBsAg loss and undetectable HBV DNA. When immune responses are misdirected, multisystem toxicity occurs. This includes immune-mediated hepatitis, autoimmune thyroiditis, cytopenias, neuropsychiatric symptoms, and barrier tissue inflammation. This paradox, wherein therapeutic and adverse effects represent opposing faces of the same dysregulated immune activation, lies at the core of PegIFN-α therapy. ALT, alanine aminotransferase; Bregs, regulatory B cells; CNS, central nervous system; DCs, dendritic cells; HBV, hepatitis B virus; HBsAg, hepatitis B surface antigen; HSPCs, hematopoietic stem and progenitor cells; IFN-α, interferon-alpha; IFNAR, interferon-α/β receptor; IFN-γ, interferon-gamma; IL, interleukin; ISGs, interferon-stimulated genes; JAK-STAT, Janus kinase-signal transducer and activator of transcription; NK cells, natural killer cells; PegIFN-α, pegylated interferon-alpha; Th1, T helper 1 cells; Tfh, follicular helper T cells; TNF-α, tumor necrosis factor-alpha; TPO, thyroid peroxidase; Tg, thyroglobulin; Tregs, regulatory T cells.

The clinical challenge of PegIFN-α therapy stems from a foundational immunological paradox, in that its therapeutic and toxic mechanisms are intrinsically inseparable. This arises because PegIFN-α activates a broad, systemic immunostimulatory program that lacks the specificity to distinguish between virus-infected and healthy host tissues. Upon binding to the interferon-α/β receptor (IFNAR), PegIFN-α triggers JAK-STAT signaling, orchestrating a concerted immune response. This includes: (1) a pro-inflammatory cytokine milieu; (2) generalized activation of innate and adaptive immune cells; (3) ubiquitous upregulation of antigen presentation; and (4) disruption of immunoregulatory checkpoints. Collectively, these changes constitute PegIFN-α-induced systemic immune activation, a broad, often dysregulated state of host immunity that underlies both therapeutic and toxic effects. This powerful but indiscriminate immune thrust is essential for purging infected hepatocytes, yet simultaneously becomes the instrument of collateral tissue damage. As visualized in **Figure 1** and summarized in **Table 2**, this dysregulated immune activation unfolds via interconnected pathogenic pathways. The following subsections dissect how it manifests across different systems, culminating in the organ-specific toxicities detailed in Section 2. It is crucial to distinguish these *de novo*, therapy-induced immunopathologies from the pre-existing T-cell exhaustion characteristic of chronic HBV, a state that PegIFN-α aims to reverse but may, in fact, insufficiently address or even paradoxically exacerbate.

**Table 2:** Key host immunopathological pathways in PegIFN-α therapy for chronic hepatitis B: an integrated framework.

Mechanism category	Key cells/tissues involved	Mechanism type	Core signaling pathways & molecular drivers	Critical effector molecules & processes	Pathophysiological consequence/clinical correlation	Key references
Systemic Inflammation & Cytokine Release	Macrophages, dendritic cells, endothelial cells.	Immune-mediated	Synergistic TNF-α & IFN-γ → hyperactivated JAK/STAT1; NF-κB activation.	Excessive TNF-α, IL-6, IFN-γ production; self-amplifying feedback loop; SASP induction.	Early toxicities: Flu-like syndrome (fever, myalgia, fatigue). Propagates tissue injury systemically.	(36–38)
Hematopoietic Suppression & Dysfunction	HSPCs, myeloid progenitors, megakaryocytes.	Direct cellular toxicity	Chronic JAK-STAT → SOCS induction; p38 MAPK activation.	SOCS-1-mediated inhibition of thrombopoietin signaling; HSPC exhaustion & skewed differentiation; ineffective hematopoiesis.	Dose-limiting cytopenias (neutropenia, thrombocytopenia); ↑ infection/bleeding risk.	(18, 39–42, 44)
Paradoxical Lymphocyte Consumption	Circulating CD8^+^ T cells (total & virus-specific).	Direct cellular toxicity (on immune cells)	PegIFN-α–potentiated pro-apoptotic cytokine milieu.	Activation-induced cell death/dysfunction; failure to reconstitute exhausted HBV-specific clones.	Systemic lymphopenia; highlights disconnect between innate activation and adaptive immune reconstitution.	(13, 94)
Loss of Peripheral Tolerance & Autoimmunity	APCs, Tregs, autoreactive lymphocytes.	Immune-mediated	IFN-α → IRAK1-p38 MAPK pathway (Treg apoptosis); JAK-STAT-driven MHC I/II upregulation.	IRAK1-mediated Treg apoptosis; enhanced self-antigen presentation; autoantibody production (e.g., anti-TPO).	Autoimmune phenomena: Thyroid dysfunction (most common), drug-induced autoimmune hepatitis.	(22–24, 50, 51, 95)
Neurotoxicity via Central Inflammation	Microglia, neurons, HPA axis.	Immune-mediated	Peripheral cytokines → CNS JAK-STAT/IDO activation; HPA axis dysregulation.	IDO-mediated tryptophan/serotonin depletion; dopaminergic dysfunction; sustained microglial activation.	Neuropsychiatric toxicity: depression, cognitive impairment, fatigue.	(19, 45–49)
Pulmonary Vascular Injury	Pulmonary endothelium, vascular smooth muscle cells.	Direct cellular toxicity	Sustained IFN-α → Caveolin-1 insufficiency → dysregulated eNOS/STAT1/AKT; chemokine induction.	Endothelial dysfunction (↑ CX3CL1); constitutive STAT1 activation; interferon gene signature.	Pulmonary arterial hypertension (PAH); interstitial inflammation.	(68, 69, 71–73)
Cardiac & Renal Direct Immunotoxicity	Cardiomyocytes, cardiac endothelium; renal podocytes/endothelium.	Cardiac: Immune-mediated	Cardiac: Cytokine-mediated negative inotropy. Renal: Direct IFN-α/β signaling.	Cardiac: TNF-α/IL-1β-mediated contractile depression. Renal: Podocyte injury, endothelial thromboresistance impairment.	Cardiac: Rare myopericarditis, cardiomyopathy. Renal: Proteinuria, FSGS, thrombotic microangiopathy.	Cardiac (74–76, 80, 82):
		Renal: Direct cellular toxicity				Renal (84, 86–88, 91):
Gastrointestinal & Dermatological Toxicity	Gut epithelium, skin keratinocytes/lymphocytes.	GI: Direct cellular toxicity	GI: Synergistic IFN-γ/TNF-α → JAK/STAT1 cytotoxicity. Derm: Direct IFN-α signaling on immune cells.	GI: Cytokine-driven sickness behavior, epithelial barrier disruption. Derm: Hair cycle disruption (alopecia), psoriasiform eruptions.	GI: Nausea, anorexia, diarrhea. Derm: Alopecia, rash, psoriasis exacerbation.	GI (54–56, 96):
		Derm: Immune-mediated				Derm (57–60):
Ocular Vascular Injury	Retinal capillary endothelium, platelets.	Direct cellular toxicity	Cytokine-mediated endothelial injury (JAK/STAT, NF-κB); IL-17A activation of JAK1.	Endothelial damage/permeability (TNF-α, IFN-γ, IL-17A); synergy with thrombocytopenia.	Asymptomatic retinopathy (common); symptomatic hemorrhage (rare).	(27, 30, 31, 61, 62)

This table summarizes the principal immunopathological pathways discussed in Section 3. The “Mechanism Type” column distinguishes between Immune-mediated toxicities (where tissue damage is inflicted by activated immune effector cells such as T cells, NK cells, or autoantibodies) and Direct cellular toxicity (where the JAK-STAT signaling cascade directly induces dysfunction, stress responses, or apoptosis in the affected non-immune target cells). Both categories are direct consequences of PegIFN-α-induced systemic immune activation, which underlies both therapeutic efficacy and multisystem toxicity. These stand in contrast to the pre-existing state of T-cell exhaustion characteristic of chronic HBV infection, which the therapy aims to reverse. The central clinical paradox lies in attempting to overcome the latter without overwhelming the host via the former.

AKT, protein kinase B; APC, antigen-presenting cell; CD8, cluster of differentiation 8; CX3CL1, C-X3-C motif chemokine ligand 1; eNOS, endothelial nitric oxide synthase; FSGS, focal segmental glomerulosclerosis; GI, gastrointestinal; HBV, hepatitis B virus; HPA, hypothalamic-pituitary-adrenal; HSPC, hematopoietic stem and progenitor cell; IDO, indoleamine 2,3-dioxygenase; IFN, interferon; IL, interleukin; IRAK1, interleukin-1 receptor-associated kinase 1; JAK, Janus kinase; MAPK, mitogen-activated protein kinase; MHC, major histocompatibility complex; NF-κB, nuclear factor kappa-light-chain-enhancer of activated B cells; PAH, pulmonary arterial hypertension; PegIFN-α, pegylated interferon-alpha; SASP, senescence-associated secretory phenotype; SOCS, suppressor of cytokine signaling; STAT, signal transducer and activator of transcription; TNF-α, tumor necrosis factor-alpha; Treg, regulatory T cell.

### 3.1 Immune-mediated inflammatory response and cytokine release

The cascade is initiated by PegIFN-α binding to the type I interferon receptor (IFNAR), activating the JAK-STAT pathway in key innate and adaptive immune cells (36). In macrophages and dendritic cells, which serve as key sentinels of the innate immune system, this signaling drives a transcriptional program essential for antiviral immunity, including maturation, enhanced antigen presentation, and production of pro-inflammatory cytokines (36, 97, 98). While this response is intended to be localized and controlled to focus immune attack on HBV-infected hepatocytes, the sustained and systemic nature of PegIFN-α exposure frequently transforms it into a dysregulated, self-amplifying state of systemic inflammation and cytokine release.

This inflammatory cascade is exemplified by studies showing that exogenous IFNα can directly modulate macrophage phenotypes and functions via this signaling axis, such as inducing M1 polarization through the JAK-STAT-dependent hepcidin-ferroportin axis, a mechanism demonstrated in the context of HBV infection (97). The potent inflammatory response is fueled primarily by the hyperactivation of innate immune sentinels, with the myeloid lineage serving as a major cellular target. PegIFN-α acts as a potent activator of dendritic cells, driving their maturation, boosting antigen presentation, and triggering pro-inflammatory cytokine production (98). Critically, in patients undergoing PegIFN-α therapy for chronic hepatitis B, this immunostimulatory milieu fosters a potent Th1 response, which in turn drives the polarization of intrahepatic macrophages toward an M1 phenotype, a process directly linked to successful HBsAg clearance (99). This polarization, mediated by sustained type I interferon signaling, is also accompanied by profound metabolic reprogramming that may predispose these cells to a pathogenic state (100–102). The collective activation markedly elevates the secretion of cytokines such as TNF-α, IL-6, and IL-1β. IFN-α itself acts as a potent pro-inflammatory and pro-apoptotic signal, directly sensitizing tissues to immune-mediated injury. Studies in epithelial cells demonstrate that IFN-α primes them for TNF-α-mediated cytotoxicity (55), thereby igniting a self-reinforcing loop. The resultant overproduction of effector cytokines, chiefly TNF-α, IL-6, and IFN-γ, creates a pathogenic positive feedback circuit that amplifies local inflammation into a systemic inflammatory cascade (37, 38). In particular, the synergistic action of TNF-α and IFN-γ potently hyper-activates the JAK/STAT1 pathway, establishing a pervasive inflammatory state. Beyond acute inflammation, this sustained signaling can induce a senescence-associated secretory phenotype in target tissues, further amplifying the local inflammatory milieu and priming tissues for immune-mediated injury (38).

This systemic inflammatory milieu underlies the characteristic early-phase toxicities. Fever arises when circulating TNF-α and IL-6 act on the hypothalamus, upregulating COX-2 and prostaglandin E2 synthesis, a final common pathway also engaged by other pyrogens. Profound fatigue and myalgia, hallmarks of the flu-like syndrome, are similarly driven by this systemic inflammatory state (92, 103).

Beyond the cytokine-driven effects of macrophages and dendritic cells, other innate lymphocyte populations also contribute to both antiviral efficacy and immunopathology. Natural killer cells, as key components of the innate immune system, exhibit a distinct mechanism of action. While they primarily exert antiviral functions through direct cytotoxicity and IFN-γ production, they may also contribute to immunopathology under certain conditions. Unlike macrophages and dendritic cells, which drive tissue damage primarily through excessive cytokine release and self-amplifying inflammatory loops, NK cell-mediated injury is more likely to result from dysregulated killing activity or excessive IFN-γ production that amplifies local inflammation. Single-cell RNA sequencing studies have identified a pro-inflammatory IFNG+ CX3CR1- NK cell subset in patients with CHB, the frequency of which positively correlates with HBsAg levels, suggesting a potential association with hepatocyte injury (104). During long-term PegIFN-α therapy, the cytotoxic subset of NK cells exhibits persistently increased activity, and this sustained hyperactivation state may elevate the risk of bystander tissue damage through perforin-granzyme-mediated killing of uninfected cells (105). Studies in HCV infection have also demonstrated that highly activated intrahepatic NK cells may contribute to ongoing liver disease, likely via IFN-γ-mediated macrophage activation and direct hepatocyte cytotoxicity (106, 107). Conversely, the therapeutic benefit of NK cell activation is evidenced by the expansion of specific cytotoxic subsets during PegIFN-α therapy, such as the CX3CR1+KLRC2- CD16hi population, which correlates with treatment response (108). Thus, NK cells exhibit a dual role during PegIFN-α therapy, promoting viral clearance when moderately activated but exacerbating tissue injury when excessively or in a dysregulated manner. This dual nature of NK cells further exemplifies the central paradox of PegIFN-α therapy, wherein the same immunostimulatory signal can yield divergent outcomes depending on the cellular context and activation intensity. Within the innate immune compartment, two distinct but complementary pathways of injury thus emerge. Macrophage- and dendritic cell-driven toxicity is primarily cytokine-mediated and systemic, whereas NK cell-associated injury is more focused and contact-dependent.

Most critically, the same inflammatory milieu that activates these innate effectors also propagates adaptive immune-mediated liver injury, laying bare the fundamental therapeutic paradox. Effector cells activated within this cytokine environment become agents of collateral damage. For example, CD8^+^ cytotoxic T lymphocytes are robustly stimulated, and their activity directly contributes to clearance of infected hepatocytes, the intended therapeutic outcome, yet inevitably causes bystander injury leading to hepatocyte death and alanine aminotransferase flares (65, 66). This functional reshaping of adaptive immunity, consistently observed in treated patients (67), underpins the antiviral effect. It is essential to distinguish these immune-mediated flares from those due to viral breakthrough, mandating vigilant HBV DNA monitoring (63, 64). Beyond the liver, persistently high cytokine levels, particularly TNF-α which correlates with symptom severity, can trigger apoptotic pathways in various tissues, illustrating the widespread collateral damage of a dysregulated immune response. This principle, where excessive cytokine activity drives immunopathology, is a hallmark not only of severe viral infections (109) but also of the iatrogenic immune state induced by PegIFN-α therapy.

In summary, the systemic inflammatory response and its resultant flu-like syndrome constitute the most immediate clinical signature of PegIFN-α-induced systemic immune activation. This pervasive inflammatory state, originating from cytokine release by activated macrophages and dendritic cells, establishes the systemic milieu that underlies and connects the diverse organ-specific toxicities explored in the following sections.

### 3.2 Bone marrow suppression and hematological toxicity

Beyond the systemic inflammatory cascade, PegIFN-α exerts a profound and paradoxical impact on the bone marrow, which serves as the primary reservoir for immune effector cells. This creates a fundamental therapeutic contradiction, wherein intense systemic immunostimulation coincides with bone marrow suppression. The resultant cytopenias, particularly neutropenia observed in over 80% of patients in real-world settings, represent a major dose-limiting toxicity (14). This toxicity stems from the direct, multi-layered action of IFN-α on the hematopoietic hierarchy.

In hematopoietic stem and progenitor cells (HSPCs), JAK-STAT activation by PegIFN-α engages a transcriptional program fundamentally different from that in hepatocytes. Transient IFN-α signaling can promote HSPC cell-cycle entry as part of an acute stress response. However, the chronic exposure mandated by therapy leads to functional exhaustion, diminished self-renewal capacity, and attrition of the long-term repopulating stem cell pool (18, 39, 40). This detrimental shift is mediated by chronic activation of stress pathways such as p38 MAPK, induction of DNA damage, and epigenetic alterations that collectively undermine stem cell fitness (39, 40).

Concurrently, persistent JAK-STAT signaling skews HSPC differentiation toward megakaryocyte-biased lineages (41). It also potently induces suppressors of cytokine signaling (SOCS) proteins, including SOCS-1, which competitively inhibit crucial pro-hematopoietic cytokine cascades such as thrombopoietin and G-CSF signaling. This directly represses the proliferation of committed progenitors and blunts compensatory feedback mechanisms (42). Further compounding this suppression, IFN-α directly compromises precursor viability by disrupting homeostatic regulatory networks and triggering cell cycle arrest and apoptosis through pathways involving TRAIL upregulation and p38 MAPK activation (43, 110).

Importantly, the clinical manifestation of “bone marrow suppression” presents a more complex picture than simple hypoplasia. A key prospective study in patients with chronic hepatitis C who developed PegIFN-α-induced neutropenia revealed that bone marrow aspirates showed active proliferation in the majority of cases (14 of 15 patients), with concurrent significant elevations in plasma G-CSF and GM-CSF (44). This observation of ineffective hematopoiesis provides a mechanistic parallel to the hematopoietic dysfunction seen in CHB (18, 39–42, 44). In this state, peripheral cytopenia coexists with compensatory bone marrow hyperplasia and elevated growth factors.

A concrete clinical example of this phenomenon is the patient with PegIFN-α-induced neutropenia who has a hypercellular bone marrow with abundant myeloid precursors but remains profoundly neutropenic, with only a transient or blunted response to G-CSF (14, 44). This dissociation illustrates that the primary defect is not in cell production, which is actually increased as seen by marrow hyperplasia, but rather in effective maturation, mobilization, or peripheral survival. This is the hallmark of ineffective hematopoiesis, distinguishing it from true aplastic anemia. Another example is interferon-induced thrombocytopenia occurring in the context of increased megakaryocytes in the bone marrow. Here, the compensatory mechanism is overwhelmed by IFN-α’s direct suppression of megakaryocyte maturation and SOCS-mediated desensitization to thrombopoietin signaling (41, 42).

Thus, PegIFN-α induces a state of hematopoietic dysfunction characterized by a dissociation between marrow cellularity and functional output, rather than pure hypoplasia. The clinical consequence is an iatrogenic state of immunodeficiency and elevated bleeding risk, a direct contradiction to the therapy’s goal of enhancing immune surveillance (14). This outcome underscores a deeper paradox. The systemic immunostimulation required for viral clearance is paradoxically sabotaged by the attrition of its very cellular source (41). Managing these dose-limiting toxicities, which necessitate vigilant monitoring and frequent dose modification or interruption, remains a central clinical challenge. This is evidenced by consistent safety profiles in trials and contemporary guidelines (14, 43, 63, 64). These findings underscore the urgent need for predictive biomarkers and adjunctive therapies that can mitigate cytopenias without compromising the essential antiviral immune thrust.

Hematological toxicity thus epitomizes the core paradox of PegIFN-α-induced immune activation. The systemic demand for immune activation is fundamentally undermined by the dysfunction and ineffective compensation of its cellular source, the hematopoietic stem and progenitor cells themselves.

### 3.3 Induction of autoimmunity

The induction of autoimmunity represents a distinct manifestation of PegIFN-α-induced systemic immune activation. The same JAK-STAT signaling that drives antiviral effects in hepatocytes triggers fundamentally different transcriptional programs in non-target tissues. In thyroid follicular epithelial cells, JAK-STAT activation by PegIFN-α potently upregulates major histocompatibility complex (MHC) class I and II molecules. This enhances the presentation of self-antigens and renders these cells susceptible to immune-mediated attack (22, 50).

This breach of peripheral tolerance is most clinically evident in the thyroid gland, where PegIFN-α induces a biphasic autoimmune thyroiditis. The initial phase is characterized by destructive thyrotoxicosis. Interferon-activated cytotoxic T lymphocytes infiltrate the thyroid, causing follicular disruption and uncontrolled release of preformed thyroid hormone. This leads to transient and often subclinical hyperthyroidism (20, 22). As the gland becomes depleted of hormone stores, the clinical picture evolves into hypothyroidism. This second phase is driven by persistent lymphocytic infiltration, cytokine-mediated inhibition of thyrocyte function through IFN-γ and TNF-α, and the production of neutralizing autoantibodies against thyroid peroxidase (TPO) and thyroglobulin (Tg) (23, 24).

This dynamic spectrum, ranging from transient hyperthyroidism to permanent hypothyroidism, results from two interconnected mechanisms. First, IRAK1-p38 MAPK-mediated apoptosis of regulatory T cells (Tregs) disrupts peripheral tolerance (51). Second, IFN-α-driven upregulation of MHC class I and II on thyroid follicular cells enhances self-antigen presentation (50, 51). Together, these processes dismantle peripheral tolerance and sustain immune-mediated attack on the thyroid.

The breach of tolerance arises from a multifaceted disruption of peripheral regulatory mechanisms. This disruption effectively induces a pronounced interferon signature similar to that observed in systemic autoimmune diseases such as lupus (22). PegIFN-α initiates autoimmunity by upregulating MHC class I and II expression, a pleiotropic immune activation mechanism driven by type I interferons (22). This upregulation on target tissues enhances self-antigen presentation and activates autoreactive CD8+ T cells (50).

Concurrently, PegIFN-α shapes the adaptive immune compartment in divergent ways. The therapy promotes the differentiation of Th1 cells, which enhance antiviral immunity, while also driving pro-inflammatory Th17 cells that contribute to autoimmune tissue injury (23, 99). At the same time, PegIFN-α directly compromises regulatory T cell stability. This Treg disruption is mediated in part through activation of the IRAK1-p38 MAPK signaling pathway, which triggers Treg apoptosis (51). PegIFN-α also impairs Treg function, collectively disrupting a critical axis of peripheral tolerance (23).

Beyond these effects on effector and regulatory T cells, PegIFN-α potently activates CD8+ cytotoxic T lymphocytes and shapes functionally distinct T cell subsets. Notably, the induction of a specific CCR7loPD-1hi follicular helper T (Tfh) cell subset has been associated with durable treatment responses, highlighting the complexity of interferon-mediated immune modulation (52, 53).

Within this broadly activated and dysregulated adaptive immune compartment, the loss of precise control creates a permissive environment for autoimmunity. Autoreactive T and B lymphocytes escape normal regulatory mechanisms. Their activation and clonal expansion culminate in the production of pathogenic autoantibodies, including anti-TPO and anti-Tg (23). This induction of thyroid autoimmunity is well documented, occurring in up to 20% of patients receiving peginterferon-alpha therapy for chronic viral hepatitis (24). Along with direct T-cell cytotoxicity, these antibodies mediate tissue injury. This cascade is central to the development of autoimmune thyroiditis, the most prevalent interferon-induced autoimmune phenomenon.

In individuals with underlying genetic susceptibility to type I interferon pathway dysregulation, often associated with risk alleles in genes such as IRF5 or STAT4, the immunostimulatory milieu created by PegIFN-α may precipitate more severe conditions (22). Cases of drug-induced autoimmune hepatitis directly attributed to interferon-alpha therapy have been documented in the literature (95, 111). These cases illustrate how a therapy designed to stimulate protective immunity can paradoxically unleash self-destructive responses.

Interferon-induced autoimmunity thus exemplifies the core therapeutic paradox of PegIFN-α therapy. The broad immune activation intended to clear the virus is directly responsible for dismantling self-tolerance. This process is initiated by JAK-STAT activation in non-target tissues such as the thyroid epithelium and is compounded by the disruption of regulatory T cell homeostasis.

### 3.4 Neuropsychiatric toxicity

The neuropsychiatric sequelae of PegIFN-α therapy arise from the propagation of peripherally derived inflammatory signals into the central nervous system (CNS). These signals, originating from the peripherally activated immune landscape, reach the CNS primarily via active transport across the blood-brain barrier or through activation of vagal afferent nerves (45). Once within the CNS, they trigger a cell type-specific response in microglia, the resident innate immune cells. While peripheral JAK-STAT activation in macrophages and dendritic cells is essential for antiviral immunity, the same inflammatory mediators engage microglia in a pathogenic program. This program includes sustained activation, production of neurotoxic mediators, and disruption of neuronal function (45, 46). This CNS-specific manifestation of systemic immune activation creates a fundamental paradox. The cytokines required for viral clearance become, within the brain, drivers of depression, cognitive impairment, and fatigue.

Within the brain, this cytokine surge disrupts fundamental neurochemical and neuroendocrine systems. A central mechanism involves the induction of indoleamine 2,3-dioxygenase (IDO). IDO shunts tryptophan metabolism away from serotonin synthesis, depleting this critical monoamine precursor (45, 47). Concurrently, inflammation perturbs dopaminergic signaling. Reduced striatal dopamine has been directly demonstrated in a primate model of chronic IFN-α exposure (48). Inflammation also induces persistent hyperactivity of the hypothalamic-pituitary-adrenal (HPA) axis, culminating in neuroendocrine dysfunction (48, 112).

Beyond acute neurochemical shifts, chronic IFN-α exposure precipitates longer-term structural and neuroinflammatory pathology. Transcriptomic analyses reveal that IFN-α disrupts synaptic signaling and activates inflammatory and metabolic pathways within key brain regions (48). Within the CNS, this peripherally initiated inflammatory signal is amplified and sustained by microglial activation. The transition of microglia to a chronically activated state perpetuates a self-sustaining neuroinflammatory environment characterized by localized production of cytokines such as IL-1β and TNF-α, along with other neurotoxic mediators (46).

This neuroinflammatory cascade translates into distinct clinical symptoms through specific pathways. Depression and anhedonia are primarily driven by cytokine-induced IDO activation, which shunts tryptophan metabolism away from serotonin synthesis and toward neurotoxic kynurenine metabolites (45, 47). Inflammation also directly impairs dopaminergic signaling, a mechanism confirmed by reduced striatal dopamine in a primate model of chronic IFN-α exposure. This underpins the profound loss of motivation and pleasure characteristic of interferon-induced depression (48, 112).

Cognitive impairment, often described as “brain fog,” arises from sustained microglial activation in the hippocampus and prefrontal cortex. This leads to suppression of hippocampal neurogenesis, potently mediated by IL-1β, and disruption of synaptic plasticity (46, 49). These structural and functional changes directly impair memory formation and executive function.

Profound fatigue during PegIFN-α therapy is a dual-origin phenomenon. Central fatigue, defined as the subjective sense of exhaustion, lack of energy, and heightened effort perception, is a direct consequence of the CNS inflammation described above. It is mediated primarily by dopaminergic dysfunction and the cytokine-induced sickness behavior syndrome (46, 112). However, it is critical to distinguish this from peripheral fatigue. Peripheral fatigue arises from therapy-induced hematological toxicity, including anemia and reduced oxygen-carrying capacity as detailed in Section 3.2, and from the systemic metabolic demands of a chronic inflammatory state. In clinical practice, these central and peripheral components often coexist and synergize, making fatigue the most common and debilitating symptom reported by patients (14, 15).

Collectively, this sustained neuroinflammatory milieu constitutes the pathophysiological substrate underlying the core neuropsychiatric sequelae of PegIFN-α therapy. It is fueled by peripheral immune dysregulation, sustained by central microglial activation, and propagated through specific neurochemical and neuroendocrine pathways. Thus, the intended peripheral immunostimulation triggers a reverberating central response, leading to a comprehensive breakdown of neurometabolic, neuroendocrine, and neuroimmune equilibrium.

In summary, neuropsychiatric toxicity arises from the propagation of peripherally initiated immune activation into the CNS. Once activated, microglia become drivers of a self-sustaining neuroinflammatory state that disrupts neuronal function and neurotransmitter balance.

### 3.5 Ocular toxicity: the case of retinopathy and hemorrhage

Interferon-associated retinopathy, though relatively uncommon, represents a clinically significant complication of PegIFN-α therapy. Patients may develop cotton-wool spots, retinal hemorrhages, and in rare cases, vision-threatening complications (28, 30). Understanding why the highly immune-privileged ocular tissue becomes vulnerable during therapy reveals a fundamental principle of PegIFN-α-induced systemic immune activation.

The pathogenesis centers on a critical synergy between two distinct processes stemming from broad immune activation, in which immune-mediated microvascular injury and therapy-induced hematological abnormalities (61).

The primary insult is vascular, driven directly by the systemic inflammatory state. In retinal capillary endothelial cells, circulating inflammatory cytokines activate JAK-STAT signaling, driving a pathogenic cascade distinct from the antiviral effects observed in hepatocytes. This cascade includes disruption of tight junctions, increased vascular permeability, and upregulation of adhesion molecules, all of which render the retinal microvasculature vulnerable to injury (31, 62). The pro-inflammatory cytokine milieu, particularly elevated TNF-α and IFN-γ produced by activated myeloid and lymphoid cells, directly damages retinal capillary endothelial cells, disrupting tight junctions and increasing vascular permeability and fragility (31). Beyond these well-characterized mediators, dysregulated immune activation may also involve upregulation of the IL-17 pathway. Experimental studies have demonstrated that IL-17A directly damages the blood-retinal barrier by activating JAK1 signaling, thereby disrupting endothelial tight junctions (62). This endothelial dysfunction, potentially compounded by immune-complex-mediated capillary occlusion, sets the stage for microvascular ischemia manifesting as cotton-wool spots and for vascular leakage (30).

Concurrently, PegIFN-α may lead to clinically significant thrombocytopenia in a subset of patients as a result of direct bone marrow suppression (14, 40, 42). The therapy impairs hematopoietic stem and progenitor cell (HSPC) function, promoting dysregulation and depletion of the stem cell pool (40). Chronic inflammatory signaling mediated by IFN-α engages stress-activated pathways such as the p38 MAPK cascade, inducing cell cycle arrest and apoptosis in hematopoietic cells and thereby contributing to the depletion of the functional progenitor pool (39). Furthermore, potent induction of suppressors of cytokine signaling, including SOCS-1, inhibits crucial pro-hematopoietic and thrombopoietic pathways (42). This collective suppression creates an iatrogenic state of impaired primary hemostasis.

When ocular-specific vascular injury coincides with thrombocytopenia, the convergence of a cytokine-injured, fragile vasculature with a deficit in platelet-mediated hemostasis dramatically escalates the risk of hemorrhage, transforming a potential microvascular insult into a sight-threatening event (61).

Prospective studies screening patients undergoing interferon therapy report a wide range in the overall incidence of retinopathy, with estimates varying from 18% to 86%. The vast majority of these cases are asymptomatic and subclinical (28). This is exemplified by studies in which over 75% of patients developed retinal changes, yet fewer than 2% experienced visual symptoms (29). Pre-existing microvascular disease, such as hypertension or diabetes, significantly increases this risk (29, 31).

Thus, ocular toxicity arises from the pathogenic convergence of two distinct outcomes of PegIFN-α-induced systemic immune activation, namely pro-inflammatory vascular damage affecting retinal capillary endothelial cells and hematopoietic suppression affecting HSPCs and platelets. This synergy vividly illustrates how the therapy’s indiscriminate immune thrust can inflict precise collateral damage on non-target tissues through interconnected mechanisms.

### 3.6 Paradoxical lymphocyte consumption and failed reconstitution

A profound and often underappreciated consequence of PegIFN-α-induced systemic immune activation is the paradoxical depletion of the very adaptive immune cells it seeks to enhance. While the therapy aims to reverse the T cell exhaustion characteristic of chronic HBV infection, longitudinal immunomonitoring reveals a significant decrease in the absolute number of circulating CD8+ T cells during treatment (13, 113). This lymphocyte consumption, distinct from the myelosuppression affecting myeloid lineages, represents a major immunologic cost of PegIFN-α therapy and directly undermines its goal of achieving functional cure.

In circulating CD8+ T cells, chronic JAK-STAT signaling within a pro-inflammatory milieu triggers outcomes fundamentally different from those observed in hepatocytes. While the same pathway in liver cells induces antiviral effectors, in persistently stimulated CD8+ T cells it drives activation-induced cell death and functional exhaustion, paradoxically depleting the cells required for viral clearance (13, 38, 113). This depletion affects both total and virus-specific populations, including CMV-specific T cells, while failing to robustly expand HBV-specific responses (13, 113).

The mechanism underlying this depletion is not direct bone marrow suppression but rather a state of chronic, high-intensity immune activation. The sustained pro-inflammatory cytokine milieu, particularly elevated TNF-α and IFN-α, drives activation-induced cell death and functional exhaustion in persistently stimulated T cells (38). This stands in stark contrast to the therapy’s intended goal of correcting the exhausted phenotype characteristic of chronic HBV infection, marked by upregulated inhibitory receptors such as PD-1 and TIGIT and impaired effector function in HBV-specific T cells (7, 94).

Clinical evidence indicates limited efficacy in achieving this goal. PegIFN-α often fails to substantially expand or functionally reconstitute exhausted HBV-specific CD8+ T cell clones (12, 94). In non-responders, persistent antigenemia and inflammation likely perpetuate this dysfunctional cycle (94). Consequently, the persistence of an exhaustion signature during therapy more accurately reflects inadequate immune reconstitution or the unresolved baseline dysfunctional state of virus-specific T cells rather than a *de novo* adverse effect (7).

Thus, the PegIFN-α response is marked by a dual failure in adaptive immunity. The therapy fails to adequately rescue the function of exhausted, virus-specific CD8+ T cells while simultaneously incurring collateral numerical depletion of the circulating CD8+ T cell pool. This dual failure underscores a fundamental therapeutic limitation. The therapy’s potent but broad innate immune thrust is insufficient to precisely rebuild a competent, pathogen-specific adaptive immune response and may actively undermine it through collateral depletion.

Importantly, the impairment of adaptive immunity extends beyond insufficient reconstitution and is actively enforced by therapy-induced immunosuppressive mechanisms. Integral to PegIFN-α-induced systemic immune activation is the expansion of regulatory immune subsets. A pivotal clinical study demonstrated that PegIFN-α-2b therapy potently expands CD24+CD38hi regulatory B (Breg) cells (12). These cells secrete IL-10 and can directly suppress the effector functions of both CD8+ T cells and NK cells, providing a direct cellular mechanism for the observed lymphocyte dysfunction (12). The clinical relevance of this finding is underscored by the inverse correlation between the frequency of these induced Breg cells and the rate of HBeAg seroconversion (12).

This mechanism exemplifies the broader paradigm wherein sustained type I interferon signaling can paradoxically establish a net immunomodulatory environment that compromises viral clearance (11). Consequently, the therapeutic ceiling of PegIFN-α may be determined not only by its capacity to stimulate immunity but equally by its propensity to induce regulatory circuits that actively suppress it. This process, centered on the depletion of CD8+ T cells and their active suppression by therapy-induced Bregs, redefines the concept of paradoxical lymphocyte consumption during therapy.

### 3.7 Pulmonary, cardiovascular, and renal toxicity: manifestations of systemic immune activation in vulnerable organs

The paradigm of PegIFN-α-induced systemic immune activation extends its reach to the pulmonary, cardiovascular, and renal systems, where it can precipitate serious, albeit less frequent, organ-specific immunopathologies. These toxicities are not incidental but represent the collateral damage of a sustained, systemic interferonogenic state impacting organs with specific vulnerabilities, particularly the vascular endothelium and sites of immune privilege. They underscore the consequence of chronic, dysregulated type I interferon signaling beyond the more common toxicity patterns.

#### 3.7.1 Pulmonary toxicity: from alveolar inflammation to pulmonary arterial hypertension

Pulmonary complications associated with PegIFN-α therapy, though rare, represent a serious form of organ-specific immunopathology. They arise from sustained JAK-STAT activation by high systemic levels of IFN-α in non-target pulmonary tissues. In pulmonary arterial endothelial cells, this signaling drives a pathogenic cascade distinct from the antiviral effects observed in hepatocytes. The cascade includes downregulation of caveolin-1 (CAV1), dysregulation of endothelial nitric oxide synthase (eNOS), and constitutive activation of STAT1 and AKT pathways (68, 69). This endothelial dysfunction, compounded by IFN-α-induced expression of pro-inflammatory chemokines such as CX3CL1, promotes vascular inflammation and remodeling, ultimately culminating in pulmonary arterial hypertension (PAH) (68, 70, 71).

The pathogenesis extends beyond a generic pro-inflammatory response to involve specific interferon-driven endotheliopathy. A key driver is the sustained, high systemic level of IFN-α induced by PegIFN-α therapy, which creates a systemic interferonogenic state that directly impacts the pulmonary compartment. The ensuing vascular pathology is a hallmark of severe toxicity, with PegIFN-α-associated PAH now formally recognized in drug-induced PAH classifications (68, 70).

The pathogenesis centers on interferon-induced endothelial dysfunction. A pivotal mechanism involves type I interferon signaling in the context of endothelial caveolin-1 insufficiency. Loss of CAV1 dysregulates eNOS, leading to constitutive activation of STAT1 and AKT pathways. This creates a vicious cycle wherein CAV1 deficiency predisposes to a type I interferon-biased inflammatory signature, and exogenous IFN-α further suppresses CAV1 expression, exacerbating endothelial dysfunction. This mechanism has been demonstrated in experimental models and human PAH tissue (69). Concurrently, IFN-α induces the expression of pro-inflammatory chemokines such as CX3CL1 (fractalkine) in pulmonary endothelial cells, promoting vascular inflammation and remodeling (71).

Clinical evidence supports the central pathogenic role of IFN-α in PAH. Elevated plasma IFN-α levels correlate with increased PAH severity and predict poorer long-term survival (71). The relevance of this pathway is further highlighted by the finding of a robust interferon gene signature in the blood of patients with various forms of PAH, as identified through transcriptomic meta-analysis (72). Diagnosis of interferon-associated PAH often occurs within the first years of therapy (68, 70), and withdrawal of IFN may lead to hemodynamic improvement, further underscoring its causative role (68).

Clinical vigilance is paramount. Any new-onset or worsening dyspnea, persistent cough, or exercise intolerance during PegIFN-α therapy mandates prompt evaluation. This should include pulmonary function tests, high-resolution computed tomography, and echocardiography. Suspicion of PAH warrants measurement of NT-proBNP and referral for right heart catheterization (68, 73).

#### 3.7.2 Cardiovascular toxicity: when systemic inflammation meets the vasculature

Cardiovascular complications associated with PegIFN-α therapy, though rare in patients without pre-existing heart disease, represent a potentially serious manifestation of systemic immune activation. These events arise when the pro-inflammatory cytokine milieu induced by therapy encounters susceptible cardiac and vascular tissues. In cardiomyocytes, cytokines such as TNF-α, IL-1β, and IL-6 exert direct negative inotropic effects, impairing contractile function (75, 76). In coronary endothelial cells, IFN-α suppresses endothelial nitric oxide synthase (eNOS) expression and bioavailable nitric oxide, leading to endothelial dysfunction (77, 78). These combined effects create a pathogenic environment that can precipitate arrhythmias, myopericarditis, and even reversible cardiomyopathy in susceptible individuals (74, 79, 80). These cardiovascular events, though rare, stem from the same pathological principles driving more common toxicities, involving a cytokine-rich milieu inflicting direct cellular injury and a breakdown of immune homeostasis that can culminate in autoreactivity.

A primary pathway involves the direct depressive effect of inflammatory cytokines on cardiac myocytes. Experimental and clinical evidence demonstrates that TNF-α, IL-1β, and IL-6, particularly in combination, exert significant negative inotropic effects on the heart (75, 76). Concurrently, these mediators drive systemic endothelial dysfunction, a critical step in increasing peripheral vascular resistance. Clinically, PegIFN-α therapy significantly impairs flow-mediated dilation, a marker of endothelial health (77). Mechanistically, IFN-α directly suppresses eNOS expression while also synergizing with other cytokines to induce potent vasoconstrictors such as endothelin-1 from vascular smooth muscle cells (78, 81).

This dysregulated, pro-inflammatory, and vasoconstrictive environment sets the stage for more severe immune-mediated injury. Clinical reports have documented a spectrum of cardiovascular events, including arrhythmias, myopericarditis, coronary vasculitis, and reversible cardiomyopathy (74). These events can be understood as severe manifestations of the loss of peripheral tolerance discussed in Section 3.3, wherein autoreactive responses target cardiac antigens, or as consequences of intense local inflammation and cytotoxicity. For example, the pro-inflammatory cytokine milieu characteristic of PegIFN-α therapy can sustainably activate pro-apoptotic pathways in cardiomyocytes via transcription factors such as STAT1 and NF-κB, as demonstrated in models of inflammatory myocardial injury (79).

Emerging evidence highlights that sustained, high-magnitude type I interferon signaling can inflict cardiac injury through profound immunometabolic reprogramming. Studies of endogenous interferon-inducing pathways, such as the cGAS-STING axis, reveal that type I interferon responses can disrupt NAD+ metabolism and mitochondrial function, compromising cardiac endothelial and cardiomyocyte homeostasis (80). Although this model involves IFN-β rather than IFN-α, it mechanistically underscores the broader principle that dysregulated type I interferon signaling can directly impair cardiac function. The clinical observation of reversible, interferon-associated dilated cardiomyopathy can thus be viewed as a direct manifestation of this link between profound immune activation and cardiac injury (74).

Critically, the absolute risk of significant *de novo* cardiac dysfunction in patients with chronic hepatitis B and no pre-existing heart disease appears very low. Prospective studies have shown no significant deterioration in systolic function during therapy (82). However, the potential severity of these events, particularly in individuals with subclinical cardiovascular disease whose vasculature and myocardium may be primed for injury, mandates caution. A careful baseline cardiovascular assessment and vigilant monitoring for new symptoms such as palpitations, chest pain, undue fatigue, or edema throughout treatment remain essential components of managing PegIFN-α-induced systemic immune activation (63).

#### 3.7.3 Renal toxicity: the spectrum of interferon-related nephropathies

The kidney is a recognized target of systemic immune dysregulation induced by PegIFN-α therapy, giving rise to a broad spectrum of glomerular and vascular pathologies (83, 85). These renal complications, though less common than hematological or neuropsychiatric effects, can be severe and potentially irreversible. The pathogenesis is multifactorial, stemming from the same dysregulated immune activation detailed throughout this review, with key mechanisms including systemic autoimmunity (Section 3.3), complement activation, direct endothelial cytotoxicity, and podocyte injury driven by sustained type I interferon signaling.

In renal podocytes, sustained type I interferon signaling triggers cytoskeletal disruption, apoptosis, and loss of slit diaphragm integrity (86, 87). In glomerular endothelial cells, the same signaling impairs thromboresistance by disrupting angiogenesis and fibrinolysis (88). These podocyte- and endothelium-specific injuries underlie the broad spectrum of interferon-associated nephropathies, ranging from asymptomatic proteinuria to collapsing focal segmental glomerulosclerosis (FSGS) and thrombotic microangiopathy (TMA) (83–85, 89).

The most well-characterized lesion associated with interferon therapy is collapsing FSGS, which has been robustly linked to treatment with IFN-α, IFN-β, or IFN-γ (84). However, the clinical spectrum extends beyond this entity to encompass membranous nephropathy, minimal change disease, and TMA (83, 84, 89). *In vitro* evidence demonstrates that IFN-α and IFN-β impair endothelial cell functions critical for thromboresistance, including angiogenesis and fibrinolysis, providing a mechanistic basis for TMA development (88). Additionally, cytokines such as IFN-γ and IL-4 have been shown to be directly toxic to cultured podocytes, disrupting monolayer integrity (86).

Beyond direct cytotoxicity, IFN-α/β signaling drives podocyte injury through multiple interconnected pathways. It promotes podocyte loss and suppresses regenerative capacity, contributing to glomerulosclerosis (87). Simultaneously, it activates protective autophagy as an adaptive cellular response to mitigate damage, though this mechanism is often insufficient to prevent progressive injury (90, 91).

Clinically, presentation ranges from asymptomatic non-nephrotic proteinuria to full nephrotic syndrome, hematuria, and acute kidney injury. Withdrawal of PegIFN-α is the cornerstone of management and can lead to remission in many cases. However, progression to end-stage renal disease, particularly in severe presentations such as TMA, has been reported (89).

Proactive renal monitoring is therefore essential. Baseline assessment must include serum creatinine, estimated glomerular filtration rate (eGFR), and urinalysis with microscopy. These parameters should be checked regularly during treatment, typically every 4 to 12 weeks. The emergence of new or increased proteinuria (exceeding 1+ on dipstick), an active urinary sediment, or an unexplained rise in serum creatinine (greater than 30% from baseline) warrants prompt nephrological evaluation (63, 83).

Pulmonary, cardiovascular, and renal toxicities, though less frequent than hematological or neuropsychiatric effects, represent severe manifestations of PegIFN-α-induced systemic immune activation. They occur when the chronic, systemic interferon signal and associated inflammation encounter specific organ vulnerabilities, particularly vascular endothelial dysfunction in pulmonary and renal vessels and coronary arteries, and breakdown of local immune tolerance in podocytes and cardiomyocytes. Their pathogenesis often involves positive feedback loops that amplify local interferon production or injury. These serious toxicities underscore the far-reaching consequences of therapeutic immune activation and reinforce the imperative for vigilant, organ-specific monitoring and early intervention in at-risk patients.

### 3.8 Involvement of host factors in other systemic reactions

The pervasive nature of PegIFN-α-induced systemic immune activation ensures that few organ systems remain unaffected. Beyond the major toxicities previously detailed, this dysregulated immune state frequently manifests as more common, though typically less severe, gastrointestinal and dermatological disturbances. These reactions serve as direct clinical signatures of the sustained systemic inflammatory milieu.

#### 3.8.1 Gastrointestinal system

Gastrointestinal disturbances, including nausea, anorexia, and diarrhea, are common during PegIFN-α therapy, affecting an estimated 15% to 40% of patients (54, 55). These symptoms arise from both indirect and direct effects of circulating pro-inflammatory cytokines.

Through indirect pathways, cytokines such as TNF-α, IL-1β, and IFN-γ induce sickness behavior characterized by anorexia and nausea (54, 55). In parallel, the same cytokine milieu exerts direct effects on the gastrointestinal tract. At the functional level, these mediators dysregulate gut motility and secretion (54–56).

At the cellular level, in intestinal epithelial cells, JAK-STAT activation by circulating cytokines triggers pathogenic cascades distinct from those observed in hepatocytes. Synergistic signaling by IFN-γ and TNF-α induces caspase-8-dependent cell death through JAK-STAT pathways, directly compromising epithelial viability (56, 96). Concurrently, IL-1β increases tight junction permeability, disrupting epithelial barrier integrity (56, 96). Although overt barrier disruption may represent a later event, these direct cytotoxic and functional effects contribute to the early gastrointestinal symptoms observed during therapy.

#### 3.8.2 Dermatological system

Dermatological reactions are common during PegIFN-α therapy and manifest in several forms, including alopecia, psoriasiform eruptions, and injection-site reactions (57–60). These cutaneous manifestations directly reflect the broad immune activation induced by therapy, wherein a type I interferon-rich environment triggers cell type-specific programs in non-target skin tissues.

Alopecia results from cytokine-mediated disruption of the hair follicle cycle, specifically anagen effluvium, which leads to temporary hair loss (57–60). This process is driven by the same inflammatory mediators that underlie systemic immune activation.

In keratinocytes and cutaneous lymphocytes, the type I interferon-rich environment induced by PegIFN-α triggers pathogenic cascades distinct from those observed in hepatocytes. IFN-α itself acts as a potent inducer of psoriatic inflammation by activating cutaneous lymphocytes, leading to psoriasiform eruptions (57, 58). This activation creates an inflammatory milieu often characterized by dysregulation of local immune axes, particularly the Th17/Treg balance, which underpins the spectrum of interferon-associated inflammatory skin disorders (59, 60).

Injection-site reactions are localized inflammatory responses to the foreign protein and typically manifest as erythema, swelling, or discomfort at the site of administration. Unlike the systemic cutaneous reactions described above, these are confined to the injection site and reflect a local immune response to the pegylated interferon formulation.

Together, these dermatological reactions illustrate how systemic immune activation extends beyond primary target organs to affect the skin through both direct cytokine effects on keratinocytes and local immune dysregulation involving cutaneous lymphocytes.

#### 3.8.3 Musculoskeletal system

Musculoskeletal symptoms, particularly myalgia and arthralgia, are cardinal features of the interferon-induced flu-like syndrome and affect the majority of patients during PegIFN-α therapy (37, 92). These symptoms are driven predominantly by cytokine-mediated activation of nociceptive pathways, with IL-6 and TNF-α playing key roles in generating pain and discomfort (37, 92).

Beyond these common and generally self-limited symptoms, PegIFN-α can, in rare instances, trigger more serious musculoskeletal complications. In susceptible individuals, the therapy may precipitate *de novo* autoimmune inflammatory myopathies or exacerbate pre-existing autoimmune rheumatic diseases such as rheumatoid arthritis (22). These serious manifestations stem from PegIFN-α’s fundamental ability to disrupt immune tolerance and promote a pro-inflammatory adaptive immune shift, a mechanism that is not tissue-specific but can manifest in susceptible musculoskeletal tissues.

In musculoskeletal tissues, including myocytes, synovial cells, and nociceptive neurons, the pro-inflammatory cytokine milieu induced by PegIFN-α triggers responses distinct from those observed in hepatocytes. While the same inflammatory mediators contribute to viral clearance in the liver, in the musculoskeletal system they activate pain pathways and, in predisposed individuals, can initiate or exacerbate autoimmune inflammation (22, 37, 92).

Management of these conditions requires clinical discernment. Common myalgia responds to symptomatic treatment, while suspected autoimmune complications warrant rheumatologic evaluation and possible therapy modification.

In summary, gastrointestinal, dermatological, and other systemic toxicities are not minor ancillary effects. They are direct clinical readouts of the underlying PegIFN-α-induced systemic immune activation. These manifestations exemplify how the therapy’s indiscriminate immunostimulatory thrust, while aimed at the liver, inevitably propagates systemic inflammatory signaling that can disrupt homeostasis in virtually all organ systems. The effects extend to intestinal epithelial cells, keratinocytes, cutaneous lymphocytes, and musculoskeletal tissues. This continuum of tissue injury, from the liver to the bone marrow, the central nervous system, the skin, the gut, and beyond, ultimately stems from a single immunological cascade. It highlights the inherent challenge of achieving targeted antiviral immunity without incurring systemic immunopathological costs.

This mechanistic analysis establishes a central tenet. PegIFN-α-associated adverse reactions are not incidental side effects but direct, predictable consequences of its primary mechanism, namely PegIFN-α-induced systemic immune activation. The inextricable link between the immunostimulation required for efficacy and the immunopathology that causes toxicity mandates a sophisticated, dual-pillar strategy in clinical management. Grounded in the pathophysiology detailed above, this strategy comprises two components. The first is pre-therapy risk prediction through profiling of individual susceptibility. The second is on-treatment navigational management employing dynamic, evidence-based interventions to preserve a viable therapeutic window. The following section translates this mechanistic framework into contemporary practice. It outlines structured monitoring, graded management algorithms, and supportive care designed to navigate this dichotomy, optimize safety, and preserve the opportunity for a functional cure.

## 4 Predictive biomarkers and management strategies

### 4.1 Pharmacological distinctions between PegIFN-α-2a and PegIFN-α-2b and their clinical implications

The two clinically available subtypes of PegIFN-α, PegIFN-α-2a and PegIFN-α-2b, differ in their polyethylene glycol (PEG) conjugation chemistry and resultant pharmacokinetic profiles. These differences dictate their distinct dosing regimens and may influence the kinetics or intensity of some adverse effects. A clear understanding of these pharmacological distinctions is essential for interpreting the mechanistic basis of toxicity and for optimizing clinical management.

PegIFN-α-2a is produced by conjugating a single branched 40-kDa PEG chain to the interferon-α-2a protein via a stable amide bond, resulting in a molecule with an approximate molecular weight of 60 kDa (32). In contrast, PegIFN-α-2b consists of a linear 12-kDa PEG chain conjugated to interferon-α-2b via an unstable urethane linkage, yielding a smaller molecule of approximately 31 kDa (32). These structural differences significantly impact the volume of distribution, proteolytic stability, and systemic clearance of each subtype.

The larger, branched PEG moiety of PegIFN-α-2a restricts its volume of distribution largely to the vascular compartment. This results in a longer elimination half-life of approximately 80 hours and sustained serum concentrations, which supports a fixed-dose regimen of 180 μg weekly (32, 33). Conversely, the smaller, linear PEG chain of PegIFN-α-2b allows wider distribution into extravascular tissues. This leads to a shorter half-life of approximately 40 hours and greater inter-individual variability in exposure. Consequently, PegIFN-α-2b is dosed based on body weight at 1.5 μg/kg weekly to account for this variability (32–34). Both subtypes are cleared primarily via hepatic metabolism and renal excretion, though the specific clearance pathways differ due to their distinct molecular sizes and PEG configurations.

Despite these pharmacokinetic differences, both subtypes exert their therapeutic effects through the same mechanism, namely binding to the interferon-α/β receptor (IFNAR) and activating the JAK-STAT signaling cascade. This shared upstream molecular trigger logically predicates a common framework of systemic immune activation and dysregulation as the basis for both efficacy and toxicity. Direct comparative studies of the two subtypes in chronic hepatitis B are limited, but available data suggest comparable efficacy and adverse event rates (35). More extensive evidence from chronic hepatitis C populations, where both subtypes have been widely used in combination with ribavirin, consistently demonstrates similar incidences of class-defining toxicities. These include flu-like symptoms, hematological abnormalities such as neutropenia and thrombocytopenia, neuropsychiatric events, and thyroid dysfunction (33, 34). These observations support the concept that the adverse effect profile is a fundamental class characteristic of PegIFN-α therapy rather than a subtype-specific phenomenon. Recognition of this class effect is clinically important, as it validates the application of a unified monitoring and management framework to patients receiving either PegIFN-α subtype.

Therefore, while the pharmacological distinctions between PegIFN-α-2a and PegIFN-α-2b inform their respective dosing schedules and may influence the kinetics of certain adverse effects, the monitoring and management principles elaborated in this review are conceptually applicable to therapy with either subtype. In clinical practice, dose adjustments must adhere to the specific product’s prescribing information. Illustrative examples include reduction from 180 μg to 135 μg weekly for PegIFN-α-2a and from 1.5 μg/kg to 1.0 μg/kg weekly for PegIFN-α-2b. It is essential to distinguish this immunostimulatory toxicity profile, which represents a PegIFN-α class effect, from the immune-related adverse events of checkpoint inhibitors. The latter arise primarily from disinhibition of autoreactive lymphocytes, underscoring the necessity for mechanism-specific management strategies. The key pharmacological and clinical characteristics distinguishing PegIFN-α-2a from PegIFN-α-2b are summarized in **Table 3**.

**Table 3:** Pharmacological and clinical characteristics of PegIFN-α-2a and PegIFN-α-2b.

Characteristic	PegIFN-α-2a	PegIFN-α-2b
PEG structure	Branched 40-kDa	Linear 12-kDa
Molecular weight	~60 kDa	~31 kDa
Half-life	~80 hours	~40 hours
Volume of distribution	Largely vascular	Wider tissue distribution
Dosing regimen	Fixed dose: 180 μg weekly	Weight-based: 1.5 μg/kg weekly
Primary clearance	Hepatic + renal	Hepatic + renal
Key references	(32, 35)	(32–35)

Data compiled from (32–35). Half-life values represent population estimates and may vary between individuals.

PEG, polyethylene glycol; kDa, kilodalton; μg, microgram; wk, week.

### 4.2 Risk prediction and personalized management

Building on this understanding of the pharmacological distinctions and shared toxicity profile of PegIFN-α, effective clinical management requires strategies to both preemptively identify high-risk individuals and actively manage the resultant immune imbalance. Risk prediction begins with comprehensive profiling of baseline host factors that collectively determine an individual’s susceptibility to the adverse effects of systemic immune activation. Key considerations include immunogenetic polymorphisms that may influence resilience or susceptibility to systemic immunoinflammatory stress.

Although direct genetic associations with PegIFN-α toxicity in chronic hepatitis B are not fully established, insights can be drawn from polymorphisms linked to dysregulation of key pathways targeted by the therapy. For instance, variations in genes controlling inflammatory tone, such as TNF and IL6, and interferon signaling fidelity, such as IFNAR1 and IFNAR2, are associated with differential risks of severe inflammation-driven liver disease progression, including cirrhosis and hepatocellular carcinoma (114–116). It is plausible that these same genetic variants may also heighten the risk of collateral tissue damage during therapeutic immune activation with PegIFN-α. This hypothesis aligns with the observation that a baseline hyper-inflammatory state predicts poorer tolerance to therapy. Although direct evidence linking these specific polymorphisms to PegIFN-α toxicity is still evolving, their established role in modulating inflammatory pathways and immune tolerance suggests they may also influence susceptibility to therapy-induced autoimmune and inflammatory side effects. A pre-treatment hyper-inflammatory state, indicated by elevated cytokines such as TNF-α or acute-phase reactants such as CRP, is genetically associated with more severe disease manifestations like cirrhosis (115). Such a state may similarly portend reduced tolerance to systemic immunostimulation, as evidenced by factors predictive of interferon dose reduction (15).

Emerging evidence from trials of novel combination regimens, such as siRNA plus PegIFN-α, further delineates the safety profile attributable to systemic immunostimulation. These studies enrich the pool of data for investigating predictive inflammatory biomarkers (117, 118). Conventional clinical parameters also play a crucial role. A history of hypertension, diabetes, or psychiatric disorders assesses specific organ system vulnerability that may be exacerbated by systemic immunopathology, as evidenced in foundational trials of PegIFN-α (16, 17). This understanding is further corroborated by contemporary studies, including those investigating novel combination regimens in adults which affirm the characteristic safety profile (43), as well as studies addressing management and monitoring needs in special populations such as children (93).

Emerging evidence also suggests that a baseline profile of immunosenescence may heighten susceptibility to PegIFN-α-induced immunopathology. This vulnerability likely stems from reduced resilience to inflammatory stress and altered immune reconstitution capacity. A key mechanism by which aging impairs innate immune precision involves disruption of the interferon regulatory factor (IRF) axis. Studies indicate that aging-associated decline in SIRT1 function results in aberrant acetylation of IRF3 and IRF7. This in turn hampers their ability to form phase-separated transcriptional condensates necessary for controlled interferon production (119). This mechanistic insight into age-associated immune dysfunction underscores the importance of considering immunosenescence in pre-therapy risk assessment. Concurrently, the aged immune landscape is often characterized by a heightened basal level of type I interferon signaling, creating a pre-existing inflammatory milieu that may be more easily tipped into a dysregulated state by exogenous interferon therapy (120).

Clinical management is therefore anchored in proactive monitoring and timely, graded intervention. The overarching goal is to maintain a viable therapeutic window, balancing antiviral efficacy against multisystem toxicity. Regular monitoring of blood counts, biochemistry, and thyroid function is mandatory, with frequency guided by the treatment phase and patient stability (121). Supportive care is essential for addressing symptomatic manifestations and maintaining treatment adherence (122, 123).

For toxicities directly attributable to PegIFN-α therapy that reflect profound disruption of immune homeostasis, specific management principles apply. In cases of severe cytopenias, significant neuropsychiatric symptoms, or ocular toxicity, dose reduction or temporary interruption forms the cornerstone of management. These interventions aim to mitigate collateral immunopathology while attempting to preserve the therapeutic window for functional cure (14, 16, 17, 43, 64, 109). Permanent discontinuation is reserved for serious, refractory, or potentially irreversible events.

**Figure 2:**
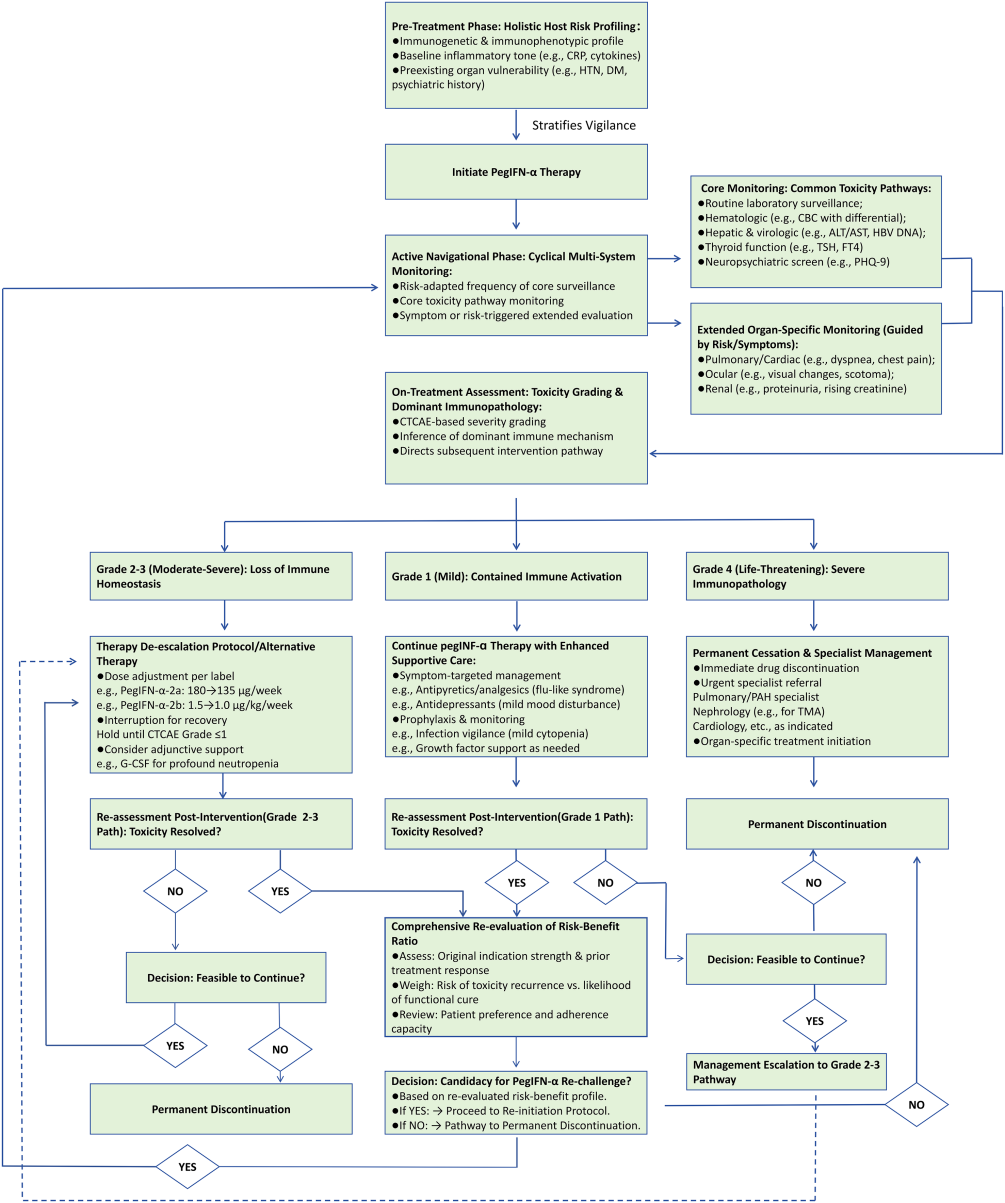
A mechanism-guided clinical management algorithm for PegIFN-α–induced immune toxicity in chronic hepatitis B This algorithm translates the framework of systemic immune activation, detailed in Section 3 and **Figure 1**, into a structured clinical pathway. It is designed to navigate the efficacy-toxicity paradox of PegIFN-α therapy. The algorithm integrates recommendations from current international guidelines, including the 2025 EASL Clinical Practice Guidelines (63), the 2018 AASLD Hepatitis B Guidance (25), and the 2022 APASL treatment algorithms (64), contextualized within the goal of functional cure (64). The algorithm follows a sequential process: pre-treatment host profiling to assess baseline susceptibility to immune-mediated toxicities; cyclical multi-system monitoring during therapy to capture dynamic changes in immune dysregulation; and on-treatment assessment, where toxicity is graded according to CTCAE v5.0. Management is then tailored to the underlying immunopathology. For Grade 1 toxicity (contained immune activation), continuation of PegIFN-α with supportive care is recommended. For Grade 2–3 toxicity (loss of immune homeostasis), therapy de-escalation through dose reduction or temporary interruption is mandated. For Grade 4 toxicity (severe immunopathology), permanent discontinuation and urgent specialist intervention are necessary. Following toxicity resolution, a comprehensive risk-benefit re-evaluation considers the strength of the original treatment indication, prior response, likelihood of achieving functional cure, and patient preference. This evaluation closes the therapeutic loop with an informed decision on continuation or re-challenge. Core monitoring parameters (complete blood count, liver function tests with ALT/AST, HBV DNA, thyroid-stimulating hormone, and PHQ-9 scores) reflect multi-guideline consensus. Absolute contraindications include decompensated cirrhosis, uncontrolled severe psychiatric illness, and pregnancy. Areas of ongoing debate are explicitly acknowledged. The optimal upper limit of normal for ALT differs between AASLD (35/25 U/L for men/women) and EASL (40 U/L for both sexes), necessitating integration of fibrosis assessment for patients in the grey zone. Interpretation of hepatitis flares remains controversial; AASLD notes that ALT elevations with declining HBV DNA may herald therapeutic immune clearance rather than drug-induced liver injury, and recent data suggest that a >1 log decline in HBsAg can help identify beneficial flares that support cautious continuation. Post-NUC cessation outcomes vary by ethnicity, with higher HBsAg loss rates in Caucasian patients but greater flare risk in Asian patients, reinforcing the need for personalized, closely monitored strategies. By synthesizing guideline-based evidence with mechanistic insights, this algorithm provides a comprehensive, evidence-based roadmap to preserve the therapeutic window for functional cure while mitigating collateral immunologic injury. Detailed monitoring parameters, frequencies, and intervention thresholds are provided in **Table 4**. This algorithm has been reviewed by hepatology experts to ensure alignment with contemporary practice standards. Abbreviations: ALT, alanine aminotransferase; AST, aspartate aminotransferase; CBC, Complete Blood Count; CTCAE, Common Terminology Criteria for Adverse Events; HBV, hepatitis B virus; LFTs, Liver Function Tests; PHQ-9, Patient Health Questionnaire-9; TSH, Thyroid-Stimulating Hormone.

Furthermore, from a holistic long-term safety perspective in chronic hepatitis B management, baseline assessment and ongoing monitoring of renal and bone parameters are imperative. This is particularly relevant when considering subsequent or concomitant long-term nucleos(t)ide analogue therapy, which constitutes the backbone of most treatment strategies. The renal and bone safety profile of long-term nucleos(t)ide analogue therapy, such as with tenofovir, has been extensively characterized (124, 125). This underscores the need for a comprehensive safety framework that accounts for cumulative and organ-specific risks across different treatment modalities and phases. A structured navigational approach to management is summarized in **Figure 2**, with detailed monitoring parameters, frequencies, and evidence-based intervention thresholds provided in **Table 4**.

**Table 4:** Clinical monitoring and evidence-based management of common PegIFN-α adverse reactions.

Adverse reaction	Baseline assessment	On-treatment monitoring	Management strategies	Key references	
Hematological Toxicity	Complete blood count (CBC) with differential.	• CBC q2–4wk for first 12–24 wk, then q4–12wk if stable.	• Neutropenia (e.g., ANC <0.75–1.0 × 10^9^/L): Reduce PegIFN dose per guideline. Consider G-CSF for profound/symptomatic cases. • Thrombocytopenia (e.g., platelets <50–70 × 10^9^/L): Dose reduction. Discontinue if platelets <25–50 × 10^9^/L or with active bleeding. • Note: Lymphopenia is managed supportively (infection prophylaxis) and is not a primary trigger for dose adjustment.	(14, 16, 17, 25, 43, 63, 64)
Thyroid Dysfunction	TSH, free T4; consider anti-TPO antibodies.	TSH and free T4 q12wk. Monitor for symptoms.	• Hypothyroidism: Initiate levothyroxine. PegIFN typically continues. • Hyperthyroidism: Symptomatic management; initiate anti-thyroid drugs if indicated. Discontinue PegIFN for severe/refractory symptoms or thyroid storm.	(20, 21, 25, 63, 64)
Neuropsychiatric Effects	Detailed psychiatric history.	• Educate patient/family. • Use validated tools (e.g., PHQ-9) at each visit.	• Mild-moderate symptoms: Supportive care ± pharmacotherapy (e.g., SSRIs). Continue with close monitoring. • Severe depression, suicidal ideation, psychosis, or mania: Immediate psychiatric consultation. Interrupt or permanently discontinue PegIFN.	(19, 25, 63, 64)
Ocular Toxicity (Vascular)	Fundoscopic exam for high-risk patients (hypertension, diabetes, pre-existing retinopathy).	• Educate to report visual symptoms immediately. • Consider periodic screening in high-risk patients. • Prompt ophthalmology referral for symptomatic patients.	• Asymptomatic, non–vision-threatening retinopathy (e.g., cotton-wool spots): May continue therapy with education/monitoring. • Symptomatic or vision-threatening findings (e.g., retinal hemorrhage): Permanently discontinue PegIFN.	(25, 27, 30, 63, 64)
Hepatotoxicity (ALT/AST Flare)	Liver function tests (LFTs); baseline HBV DNA.	• ALT/AST q4wk initially, then q8–12wk. • Critical: Monitor HBV DNA (e.g., q4–12wk) to differentiate flare etiology.	• Immune-mediated flare (ALT rise with declining/stable HBV DNA): Represents on-therapy immune activity. Usually continue with closer monitoring. • Virological breakthrough (ALT rise with rising HBV DNA): Indicates treatment failure. Adjust NA therapy; consider discontinuing PegIFN. • Severe hepatitis (ALT >10x ULN, jaundice, decompensation): Discontinue PegIFN.	(25, 63–66)
Pulmonary Toxicity (e.g., PAH)	Cardiopulmonary symptom review; assess PAH risk factors.	• Educate to report new/worsening dyspnea, chest pain, syncope, or exercise intolerance. • For symptomatic patients: Prompt evaluation with NT-proBNP and echocardiography. Specialist referral if suspected.	Confirmed PegIFN-associated pulmonary arterial hypertension mandates permanent discontinuation. Initiate PAH-specific therapy under specialist guidance.	(25, 63, 64, 68–70, 73)
Cardiovascular Toxicity	Detailed cardiovascular history, symptom review, risk assessment; baseline ECG.	Inquire about palpitations, chest pain, edema, or undue fatigue at each visit. Evaluate symptomatic patients with ECG, troponin, and echocardiography as indicated.	• Symptomatic arrhythmia, myopericarditis: Interrupt therapy for cardiology consultation. Permanent discontinuation is often required. • Asymptomatic dysfunction: Individualized risk-benefit assessment.	(25, 63, 64, 74–76, 81)
Renal Toxicity	Serum creatinine, eGFR, urinalysis with microscopy.	Monitor serum creatinine and urinalysis q12wk. Nephrology consult for unexplained creatinine rise (>30% from baseline) or new/worsening proteinuria/hematuria.	• New-onset/worsening proteinuria, hematuria, or acute kidney injury: Interrupt PegIFN and evaluate. Most toxicities are reversible upon withdrawal. • Confirmed PegIFN-induced glomerulopathy (e.g., collapsing FSGS, TMA): Permanently discontinue and manage under nephrology care.	(25, 63, 64, 83–85, 89)
Dermatological & Musculoskeletal	History of psoriasis, chronic rash, severe myalgia/arthritis.	Regularly inquire about and examine for rash, alopecia, joint pain, or muscle weakness.	• Mild rash/alopecia: Supportive care; therapy usually continues. • Severe/generalized dermatitis (e.g., psoriasis exacerbation), suspected immune-mediated myositis/arthritis: Interrupt therapy for specialist evaluation. Dose reduction or discontinuation may be necessary.	(25, 57–60, 63, 64)

This monitoring and management framework translates the mechanistic insights of systemic immune activation detailed in Section 3 into clinical practice. All recommendations are consistent with and contextualized within the principles of current international guidelines (25, 63, 64). Dose reduction and discontinuation criteria are formulation-specific. Clinical management must adhere to the latest product prescribing information and clinical practice guidelines (65, 66). Illustrative examples include: for PegIFN-α-2a, reduction from 180 µg to 135 µg weekly; for PegIFN-α-2b, reduction from 1.5 µg/kg to 1.0 µg/kg weekly. These examples are for illustration only. All decisions should be individualized based on toxicity severity, trajectory, and the specific clinical context.

ALT, alanine aminotransferase; ANC, absolute neutrophil count; anti-TPO, anti-thyroid peroxidase antibody; AST, aspartate aminotransferase; CBC, complete blood count; ECG, electrocardiogram; eGFR, estimated glomerular filtration rate; FSGS, focal segmental glomerulosclerosis; G-CSF, granulocyte colony-stimulating factor; HBV, hepatitis B virus; LFTs, liver function tests; NA, nucleos(t)ide analogue; NT-proBNP, N-terminal pro-B-type natriuretic peptide; PAH, pulmonary arterial hypertension; PegIFN, pegylated interferon; PHQ-9, Patient Health Questionnaire-9; qXwk, every X weeks (e.g., q4wk = every 4 weeks); SSRI, selective serotonin reuptake inhibitor; TMA, thrombotic microangiopathy; TSH, thyroid-stimulating hormone; ULN, upper limit of normal.

## 5 Conclusion and future perspectives

Pegylated interferon-α therapy for chronic hepatitis B embodies a fundamental immunological trade-off in clinical practice. Its potential to induce a functional cure is inextricably linked to its propensity for multisystem toxicity. Both therapeutic and adverse outcomes originate from a common source, namely the deliberate yet broad and non-specific activation of the host immune system. This review has delineated the pathogenic cascade whereby the initiating JAK-STAT signal propagates into a loss of immunological specificity. This cascade manifests clinically as a systemic inflammatory response, bone marrow suppression, a breach of self-tolerance, and a disruption of neuroendocrine-immune crosstalk. Consequently, the central clinical challenge lies in the strategic management of this potent immunostimulatory thrust to maximize antiviral efficacy while minimizing collateral host injury. Nevertheless, critical knowledge gaps persist. A deeper understanding is required regarding the precise determinants of individual susceptibility to specific toxicities, particularly autoimmunity. The potential contributory roles of factors such as the gut microbiome and baseline immunosenescence also remain to be elucidated. Addressing these questions will necessitate the integration of multi-omics data with detailed clinical phenotyping.

Future efforts should therefore pivot towards precision immunomodulation. First, advancing predictive personalization through comprehensive profiling is paramount to define an individual’s immunotype (126). Such profiling should encompass immunogenetics, transcriptomics, proteomics, and potentially the gut microbiome. Critically, this immunotypic profiling must extend beyond static markers to incorporate dynamic functional states such as immunosenescence. It should also include clinical parameters like quantitative HBsAg levels, which form the basis of contemporary treatment navigation algorithms (127). Furthermore, the systematic integration of novel virological and immunological biomarkers is essential. Evaluating cccDNA activity, treatment cessation risk, and host immune function can help construct a multidimensional framework that enables precision-driven management, particularly in the pursuit of functional cure (128). A refined understanding of the baseline immune landscape is crucial. This includes not only the presence of T-cell exhaustion, the dysfunctional state therapy aims to reverse, but also an individual’s inherent inflammatory tone and resilience to immunostimulation. This approach will enable the *a priori* identification of patients most likely to achieve a functional cure with an acceptable safety profile. Furthermore, it will guide optimal therapeutic sequencing, such as initiating nucleos(t)ide analogue therapy prior to PegIFN-α in selected candidates.

Second, the development of safer and more effective therapeutic modalities is essential. This includes agents that selectively modulate downstream inflammatory pathways, such as JAK inhibitors, to dampen toxicity while preserving antiviral signals (129, 130). It also includes novel interferons engineered for inherent cellular specificity, such as pegylated interferon-λ, which shows a dissociated safety profile (131). Moving beyond mitigating classic toxicities, the next frontier involves directly tackling the dual adaptive immune failures that limit cure rates. These failures are the inadequate rescue of exhausted T cells and their active suppression by therapy-induced regulatory circuits. On one hand, strategies are needed to enhance T-cell reconstitution. This could involve exploiting pharmacological nuances between interferon subtypes (132, 133), given the inherent limitations of type I interferon in reversing established exhaustion (134, 135). On the other hand, directly neutralizing concurrent immunosuppression is equally crucial. The discovery that PegIFN-α potently expands CD24+CD38hi regulatory B cells, which suppress T and NK cell function, offers a prime therapeutic target (12). Adjunctive strategies like anti-CD24 antibody-mediated depletion aim to precisely blunt this iatrogenic immunosuppressive arm (12). Such approaches exemplify the translational goal of disentangling interferon’s antiviral effects from its liability-associated immunomodulatory sequelae (11).

Third, innovating rational combination regimens holds significant promise. This involves optimizing existing PegIFN-α and nucleos(t)ide analogue combination strategies, such as favoring add-on over switch strategies in certain populations, to pursue higher cure rates in virologically suppressed patients. Furthermore, it necessitates addressing specific clinical challenges such as persistent low-level viremia during nucleos(t)ide analogue therapy. This state is associated with an elevated risk of fibrosis progression and hepatocellular carcinoma, and may require intensification strategies including the addition of PegIFN-α (136). Pioneering novel alliances between PegIFN-α and emerging direct-acting antivirals, such as siRNA and capsid assembly modulators, or complementary immune modulators is also crucial (137, 138). Among these, immune checkpoint inhibitors represent a particularly compelling partner. Recent clinical evidence demonstrates their capacity to reduce HBsAg and revitalize exhausted HBV-specific T cells in chronic hepatitis B (139). The rationale for combining PegIFN-α with an immune checkpoint inhibitor is profoundly synergistic. It aims to couple the broad immunostimulatory thrust of the former with the precision reversal of T-cell exhaustion afforded by the latter. This approach attacks the twin pillars of chronicity, persistent antigen and immune dysfunction, simultaneously and potentially more effectively. However, this combination embodies the apex of therapeutic ambition and immunological risk. It necessitates confronting dual-spectrum immunotoxicity, namely the dysregulated systemic immune activation characteristic of PegIFN-α and the immune-related adverse events stemming from immune checkpoint inhibitor-mediated disinhibition of autoreactive lymphocytes. Thus, the future of combinatorial immunotherapy in chronic hepatitis B hinges not merely on additive efficacy but on the successful integration of distinct toxicity management paradigms. The framework of systemic immune activation established here provides the essential mechanistic foundation for navigating PegIFN-α-associated toxicity. Therefore, a critical frontier lies in merging this framework with the well-established principles of immune-related adverse event management to design safe, effective, and truly personalized combination regimens.

The ultimate goal is to evolve towards a navigation-guided, biomarker-informed therapeutic paradigm. In this future model, the decision to employ an interferon-based strategy will be tailored by a deep, multidimensional understanding of the patient’s unique immune landscape and a sophisticated biomarker profile (128). This includes the choice of interferon type, comparing alpha versus lambda, as well as decisions about dose, duration, and optimal companion therapies. This approach will be implemented via dynamic treatment algorithms. These algorithms respond to on-treatment virological and immunological feedback, enabling the management of complex states like low-level viremia, which requires meticulous monitoring (127, 136). This strategy seeks not merely to manage the limitations of current therapy but to fundamentally resolve its core paradox. Ultimately, achieving this requires conquering the paradox of lymphocyte consumption. It demands designing interventions that vigorously stimulate antiviral immunity without incurring its debilitating systemic cost (13).

## Glossary

ALT: alanine aminotransferase
ANC: absolute neutrophil count
anti-Tg: anti-thyroglobulin
anti-TPO: anti-thyroid peroxidase
APC: antigen-presenting cell
AST: aspartate aminotransferase
CAV1: caveolin-1
CBC: complete blood count
CHB: chronic hepatitis B
CMV: cytomegalovirus
CNS: central nervous system
CRP: C-reactive protein
CTCAE: Common Terminology Criteria for Adverse Events
CTLs: cytotoxic T lymphocytes
CX3CL1: C-X3-C motif chemokine ligand 1
DC: dendritic cell
eGFR: estimated glomerular filtration rate
eNOS: endothelial nitric oxide synthase
EASL: European Association for the Study of the Liver
FSGS: focal segmental glomerulosclerosis
G-CSF: granulocyte colony-stimulating factor
GM-CSF: granulocyte-macrophage colony-stimulating factor
HBeAg: hepatitis B e antigen
HBsAg: hepatitis B surface antigen
HBV: hepatitis B virus
HPA: hypothalamic-pituitary-adrenal
HSPC: hematopoietic stem and progenitor cell
ICI: immune checkpoint inhibitor
IDO: indoleamine 2,3-dioxygenase
IFN: interferon
IFN-α: interferon-alpha
IFN-γ: interferon-gamma
IFNAR: interferon-α/β
IL: interleukin
irAE: immune-related adverse event
IRF: interferon regulatory factor
ISGs: interferon-stimulated genes
JAK-STAT: Janus kinase-signal transducer and activator of transcription (pathway)
LFTs: liver function tests
LLV: low-level viremia
MAPK: mitogen-activated protein kinase
MHC: major histocompatibility complex
NA: nucleos(t)ide analogue
NF-κB: nuclear factor kappa-light-chain-enhancer of activated B cells
NK cell: natural killer cell
NT-proBNP: N-terminal pro-B-type natriuretic peptide
PAH: pulmonary arterial hypertension
PegIFN-α: pegylated interferon-alpha
PHQ-9: Patient Health Questionnaire-9
SASP: senescence-associated secretory phenotype
siRNA: small interfering RNA
SOCS: suppressor of cytokine signaling
SSRI: selective serotonin reuptake inhibitor
STAT: signal transducer and activator of transcription
Tfh: follicular helper T
Th1: T helper 1
Th17: T helper 17
TMA: thrombotic microangiopathy
TNF-α: tumor necrosis factor-alpha
Treg: regulatory T cell
TSH: thyroid-stimulating hormone
ULN: upper limit of normal.
